# Necrological

**Published:** 1881-05

**Authors:** 


### NECROLOGICAL.

" Death is the crown of life :
Were death denied, poor man would live in vain."

Richmond, Va., Dr. Charles Macgill, an eminent physician of that city, died May 5th, 1881, in the 75th year of his age. The deceased was a native of Baltimore, and came of distinguished ancestry, being a lineal descendant of Thomas Jennings, who was king's attorney under the colonial government of Maryland, and of Rev. James Macgill, of Scotland, who settled in Maryland in 1728. He was educated at the old Baltimore College, and subsequently studied medicine and surgery under Dr. Charles G. Worthington, of Elkridge, Md., and in the Baltimore Hospital, graduating with high honors in the University of Maryland in 1828. He settled permanently in Hagerstown, Md., where he married and became a leading citizen. Before the breaking out of the late war, the sympathy of the deceased and his family being with the South, caused them to be under constant surveillance, and to suffer numerous indignities, resulting finally in Dr. Macgill's arrest and confinement as a State prisoner in Fort McHenry and other Federal prisons until November, 1862, when he was unconditionally released. The following year, during General Lee's invasion of Maryland, he cast his lot and fortunes with the Confederacy. Dr. Macgill joined the army in front of Petersburg, and remained with it until the surrender at Appomattox, doing good service in relieving the sick and wounded, and endearing himself to the entire army. After the war, Dr. Macgill made Richmond his home, where his probity and many accomplishments secured for him an extensive practice and many valued friends. He leaves an aged wife and numerous children and grandchildren. Dr. Charles Macgill, a prominent physician of Catonsville, is a son of the deceased.

Information has been received in this city of the death at Wilmington, N. C., the early part of this month, of Dr. M. J. DeRosset, formerly Professor of Chemistry in the Baltimore College of Pharmacy, and an Adjunct Professor of Chemistry in the University of Maryland. The deceased at the commencement of the war between the States was a surgeon in Bellevue Hospital at New York. He resigned, and going South, he was made an assistant surgeon P. A., C. S., and served with the artillery in Stonewall Jackson's command. He was promoted to be surgeon and inspector of hospitals for the department of Henrico, and was subsequently one of the surgeons at the Officers' Hospital in Richmond.

